# A Redefined Protocol for Protein Corona Analysis on Graphene Oxide

**DOI:** 10.1021/acsnanoscienceau.5c00052

**Published:** 2025-07-24

**Authors:** Asia Saorin, Ahmed Subrati, Alberto Martinez-Serra, Beatriz Alonso, Michael Henry, Paula Meleady, Sergio E. Moya, Marco P. Monopoli

**Affiliations:** † Department of Chemistry, 8863 Royal College of Surgeons in Ireland (RCSI), Dublin D02 YN77, Ireland; ‡ Center for Cooperative Research in Biomaterials (CIC biomaGUNE), 49562 Basque Research and Technology Alliance (BRTA), San Sebastian 20014, Spain; § 383835 GRAPHENEA SA, San Sebastian 20009, Spain; ∥ School of Biotechnology, 8818 Dublin City University (DCU), Dublin D09 W6Y4, Ireland

**Keywords:** protein corona, graphene oxide, nanosafety, protein desorption, surface interactions

## Abstract

It is well established that the biomolecular corona affects the biological behavior of nanomaterials, including cellular uptake, toxicity, and biodistribution. However, the unique physicochemical properties of advanced materials, such as graphene oxide, challenge the effectiveness of standard protocols for biomolecular corona characterization, which may lead to incomplete biomolecule recovery and biased experimental results. Protein analysis is one of the broadest techniques in the characterization of the biomolecular corona, providing important information about the composition and behavior of proteins adsorbed onto nanomaterial surfaces. Two widely accepted protein analysis methods include SDS-PAGE and mass spectrometry, and both require the complete elution of the proteins from the nanoparticle surface during denaturation steps. In this work, limitations of widely used SDS-based elution methods with GO were identified, and an improved protocol using chaotropic agents (urea and thiourea) was developed. The stepwise extraction allowed for near-complete protein desorption. Under the modified protocol, strongly bound proteins that are more hydrophobic have been proved to be underestimated using the standard method. This further reiterates the necessity for the development of methodologies tailored to the specific materials under study which accurately characterize the protein corona. Our results highlight the need for standardization and optimization of protocols to ensure reproducibility and reliability in nanosafety studies, hence promoting the safe and sustainable use of advanced materials in biological and environmental systems.

## Graphical abstract

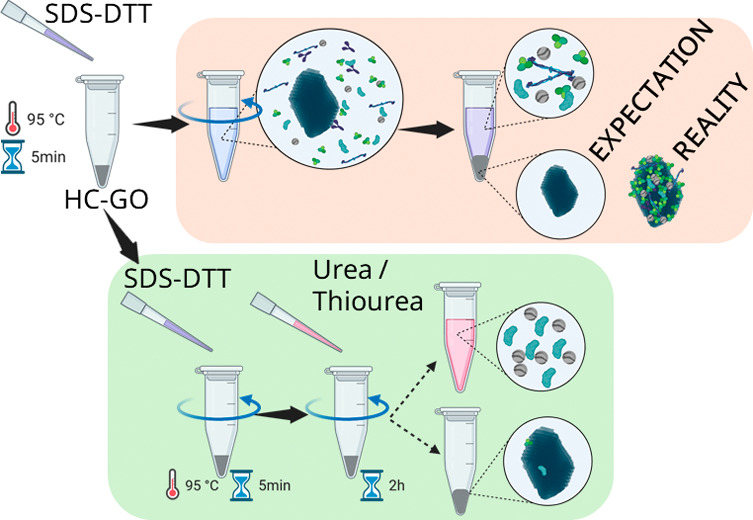

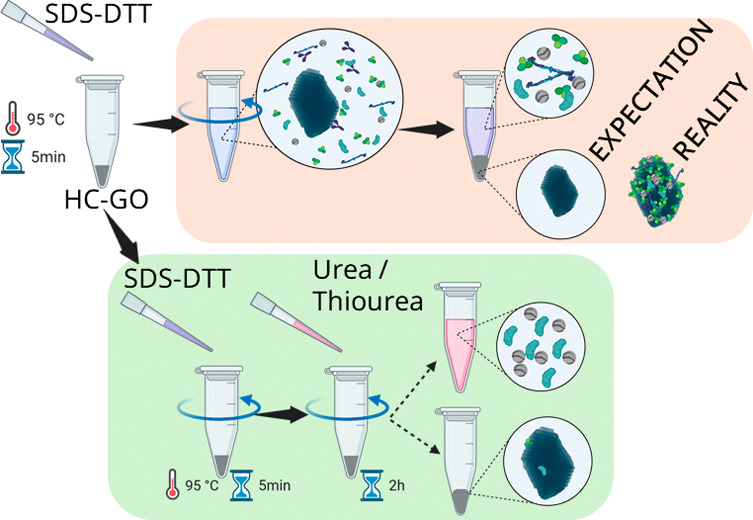

## Introduction

Graphene oxide (GO) is a single layer of graphene oxidized by the introduction of oxygen-containing functional groups, such as hydroxyls, epoxides, and carboxyls. These groups enhance GO dispersibility in aqueous environments. GO is widely investigated for bioapplications and is often incorporated in composite materials. GO has unique mechanical and electronic properties that make it very interesting for multiple industries.^1^ Due to the increasing use of graphene materials in commercial products, potential occupational and consumer exposures are also rising. Therefore, there is a need to understand their interactions with biomolecules. Recently, a standardization effort on all graphene-based derivatives has been carried to highlight the main materials’ features and their hazard profiles.^2^ In particular, studies have investigated how specific characteristics, such as average lateral dimension, number of layers, and carbon-to-oxygen ratio, influence toxicological outcomes.^2,3^ However, establishing clear correlations between GO properties and toxicity remains challenging. Even so, not only material characteristics have been found to influence toxicity and biodegradability but also the nature of molecules that are grafted on the GO surface^4,5^ and the transformation of these molecules and GO after their exposure to complex fluid. An important modality of GO interaction with biological systems involves the formation of the biomolecular corona, which is a complex layer of biomolecules formed on the nanomaterial surface after interacting with the biological environment.^6,7^ The corona introduces a new dimension to dynamic interactions between nanomaterials and their surroundings.^8^ The term protein corona (PC) is usually used instead of biomolecular corona since proteins are the most abundant bound biomolecules.^9^ Since its introduction by Linse and Dawson in 2007, the concept of PC has become extremely important in nanomedicine, nanotoxicity, and environmental science.^10–13^ The PC is usually divided into two components: the “hard corona,” (HC) composed of strongly bound high-affinity proteins that form a stable layer on top of the nanomaterial, and the “soft corona,” which contains less-affine proteins and/or proteins unable to get close enough to the nanomaterial surface due to an already formed HC, being more dynamic and participating in much more transient interactions.^14,15^ Corona formation is influenced by several factors: the physicochemical properties of the materials, the composition of the medium, and the conditions of the exposure.^16,17^ Early findings have shown that there is a correlation between corona formation and the GO-mediated cellular toxicity.^18^ Understanding the biomolecular corona is a complex task that requires an interdisciplinary approach for a comprehensive physicochemical characterization of nanoparticles and the identification of biomolecular components such as proteins, lipids, glycans, and their biological functions.

While most protocols to study the nanomaterial-biomolecular corona have been designed on spherical nanomaterials that are easily dispersible in acqueous media, in this study, we evaluated whether these protocols could be applied to advanced nanomaterials (Ad-NMs), such as GO. Our study shows that the common protocol failed to elute the strongly bound corona proteins. Indeed, GO at physiological pH presents negatively charged oxygenated functional groups along with an aromatic structure. Therefore, the GO interactions with biomolecules can occur via hydrogen bonding, electrostatic, hydrophobic van der Waals, and π–π interactions. This, combined with the high specific surface area of GO, results in the collection of very high amounts of serum proteins.^19^ More hydrophobic corona proteins required increasingly stringent conditions for their elution, such as the use of chaotropic agents^20^ suggesting the need for alternative approaches to effectively disrupt these interactions. Therefore, in this study, we report a new method specifically developed for a major and more representative protein elution from GO. The development of reliable methods for Ad-NMs is necessary for correctly estimating nano–bio interactions to safely transition these nanoparticles into industrial applications.

## Results

### Optimization of HC Extraction from GO

Three types of GO were used, each with distinct physicochemical properties. GO1 is a highly oxidized GO (48.2% oxygen by weight determined by elemental analysis) consisting of large flake sizes (*D*_50_ = 12.79 μm). GO3 was produced from GO1 by dilution of the slurry from 20 to 4 mg/mL, followed by ultrasonic treatment, which preserved the high oxidation level (48.2% oxygen by weight) while reducing the flake size (*D*_50_ = 5.97 μm). In contrast, GO2 was synthesized through a modified process to achieve a lower oxidation level (35% oxygen by weight), yielding flakes of intermediate size (*D*_50_ = 6.76 μm), comparable to GO3. In terms of particle thickness, GO1 particles retained the widest stacking of layers (*D*_50_ = 66 nm), followed by GO2 (*D*_50_ = 46 nm) and GO3 (*D*_50_ = 20 nm), reflecting the progressive exfoliation and reduction in layer stacking due to ultrasonic treatment and processing differences (Figure 1). Since GO1 and GO3 share the same extent of oxidation, their flakes appear thin and transparent with minor folds and ripples, as observed in the TEM images.^100^ Due to the relatively lower oxidation extent, flakes with a relatively higher density of defects, folds, and ripples appear in the case of GO2. These morphological features also discernibly appear in the high-resolution SEM images, where GO1 and GO3 particles exhibit flat terrains throughout, whereas GO2 particles do not as ripples and folds populate them particularly at the peripheries (Figure 1). These materials were selected in order to consider different oxidation levels and surface areas, parameters that could influence surface interaction with biomolecules. Therefore, they represent good candidates for protein corona protocol testing and development.

**Figure 1:**
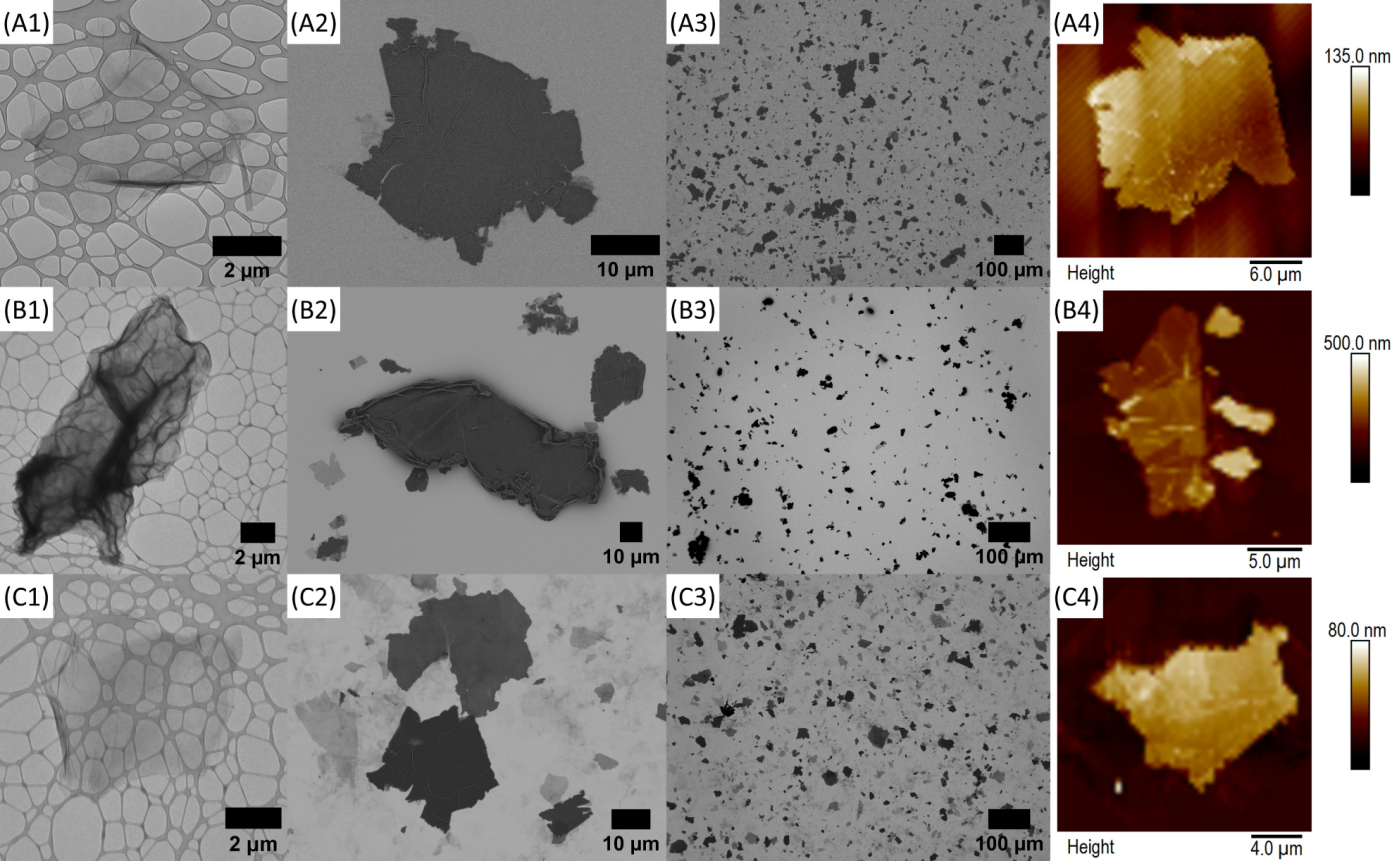
Characterization of (A) GO1, (B) GO2, and (C) GO3: (1) TEM high-resolution, (2) SEM low-resolution, (3) SEM and (4) AFM. The low-resolution SEM images were used for particle analysis to determine the values of *D*_50_ (the median particle size).

We used these GOs to test whether the common protocols for the biomolecular corona study were also applicable to these Ad-NMs. For this purpose, GOs were exposed to 10% fetal bovine serum (FBS) in phosphate-buffered saline (PBS) for 1 h to allow the biomolecular corona formation, and the GO-hard corona complexes (HC-GO) were isolated by applying centrifugation and washes as discussed in the Materials and Methods section. Prior to the SDS gel electrophoresis, HC-GO samples were treated following the commonly applied procedure (see the Standard Protein Corona Elution Protocol (STD Protocol) section). Immediately after this step, the complexes were centrifuged, and the pellet and the supernatant were separately run on SDS-PAGE to identify corona proteins that were eluted from those that were still associated with the GO material (Figures 2A and 3A).

**Figure 2:**
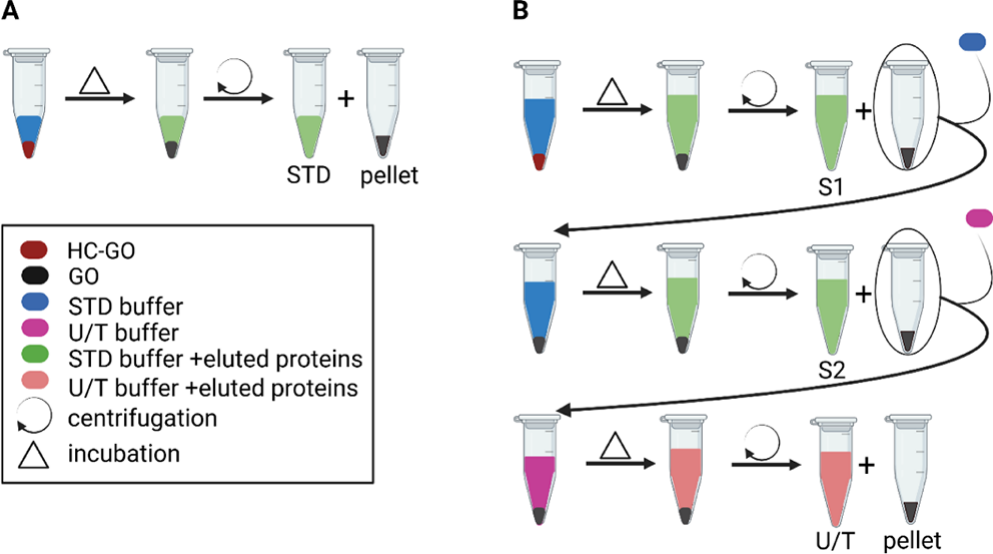
Schematic representation of the applied protocols for the elution of proteins from HC-GO. (A) Standard protocol yielding one supernatant fraction (STD) containing eluted proteins and the GO pellet. (B) Modified protocol enabling sequential collection of three supernatant fractions (S1, S2, U/T) containing eluted proteins and final GO pellet.

**Figure 3:**
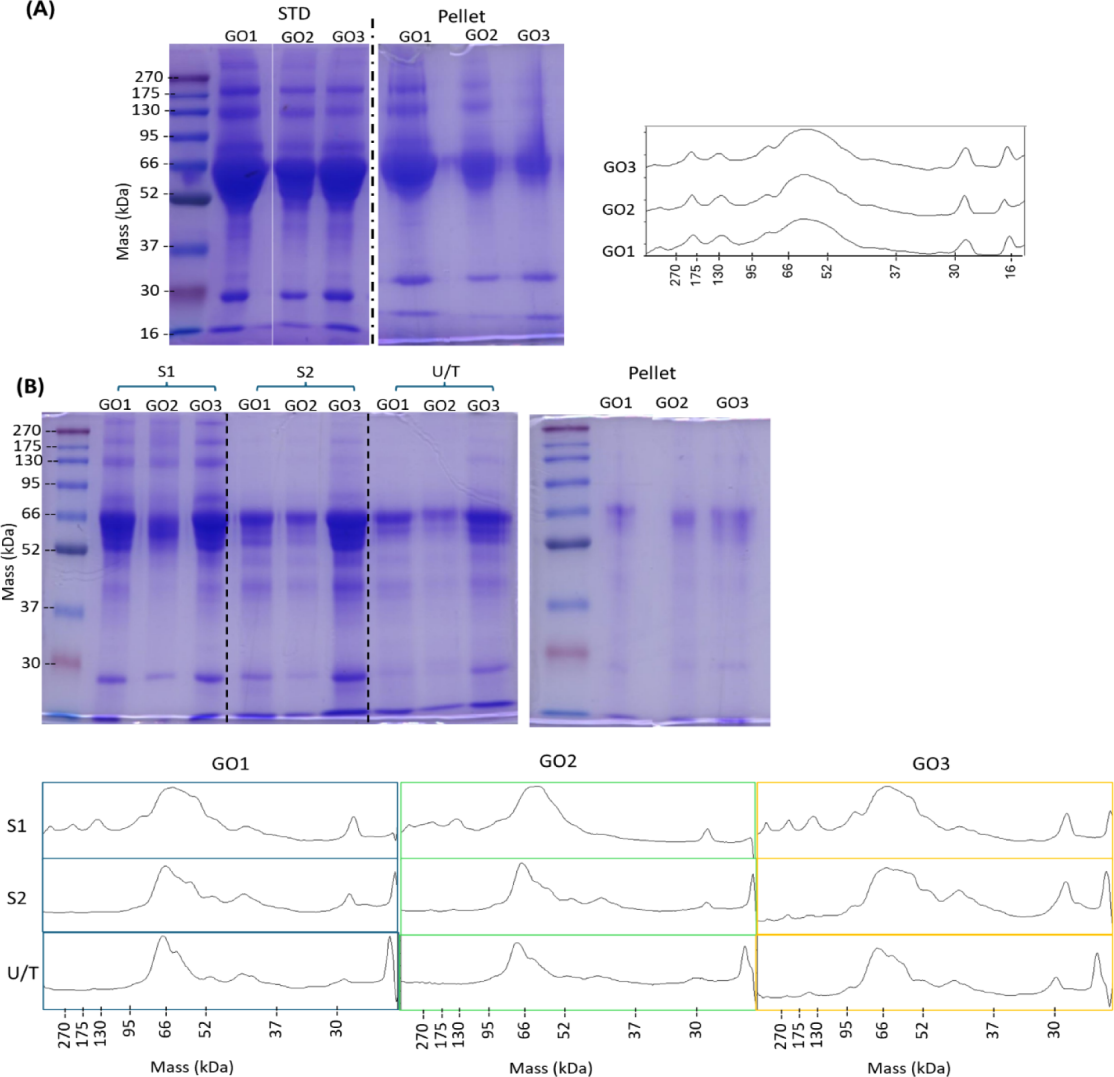
SDS-PAGE comparison of standard (STD) and modified elution protocols applied to GOs. (A) STD protocol: gel images show supernatant (STD) and corresponding pellet fractions from GO1, GO2, and GO3. Densitometric analysis of STD lanes is shown on the left. (B) Modified protocol: gel images of the three sequential extraction steps S1 (loaded volume of 3 μL), S2 (loaded volume of 15 μL), and U/T (loaded volume of 20 μL), along with corresponding pellet fractions. Densitometric analysis of S1, S2, and U/T lanes is presented below.

As shown in Figure 3A, the different GOs share a similar protein profile, but a significant amount of proteins was detected in the pellet. This finding suggested the inefficacy of the standard (STD) protocol since it failed to fully remove proteins from GOs. Therefore, GO1 was selected for further analysis as it presents big flakes and high oxidation levels, making it a good candidate for the evaluation of the corona protocol that would successfully strip off the biomolecules from the material. First, GO1 was exposed to elution buffers with increasing concentrations of SDS, varying elution volumes or sonication treatment. Variations in SDS concentration within the elution buffer led to distinct protein elution profiles, independent of buffer volume or sonication (Figure S1). In particular, a band of 66 kDa was consistently detected in all samples, while gel bands of 130 and 95 kDa were resolved at 1–2% SDS, but these bands were no longer detected at higher SDS concentrations (6–9%), suggesting that they were successfully eluted from the corona at lower SDS concentrations. This suggests that the composition of the eluted proteins does not accurately reflect the true composition of the HC, as different SDS levels elute different subsets of proteins. Moreover, regardless of the extraction volume or the addition of a sonication step, none of these elution conditions could remove proteins completely from the material surface. Therefore, different strategies were made to achieve complete protein elution from the GO1 surface using different volumes of STD buffer and repetition of the elution. Moreover, the introduction of chaotropic agents, specifically, urea and thiourea, was tested. The composition of the urea/thiourea buffer (U/T) was obtained from the literature, where it was applied for the extraction of proteins from TCA/acetone-treated smooth muscle tissues.^21^ Table S1 and Figure S2 show the different combinations of buffer and volume used to the same HC-GO1 pellet and show that a higher volume of STD buffer and the repetition of the STD elution still failed to remove full corona proteins. However, the further addition of a third elution step with U/T buffer resulted in the most effective elution protocol (Figure 2B).

As shown in Figure 3B, the protocol successfully removed the biomolecular corona from the HC-GOs as the GO pellets had a negligible gel band intensity in the SDS-PAGE gel, while each step of elution buffer exposure resulted in the release of a different set of proteins. Indeed, gel band densitometry of the different fractions highlights the differences in the eluted proteins after each elution step (Figure 3B), suggesting that different sets of biomolecules are desorbed from the corona.

The comparison of protein profiles among the different HC-GOs reveals a slightly different protein composition, as also highlighted by the SDS-PAGE analysis of S1, S2, and U/T pooled samples (Figure S3). Moreover, when these protein profiles are compared to those obtained with the STD protocol, some differences become apparent (Figures S3 and 3A). Specifically, the modified protocol yields better-resolved bands between 95 and 37 kDa, and the bands between 66 and 52 kDa appear more prominent in terms of relative intensity. This is consistent with the fact that both S2 and U/T samples show these bands as dominant. These findings further support that the modified protocol enables the extraction of a more representative protein profile.

### Mass Spectrometry Analysis of the GO-HC

Mass spectrometry was performed to get a deeper analysis of protein content in the different HC-GO1 elution fractions (S1, S2, U/T) and compare them to the proteins eluted applying the STD protocol (Figure 4 and Table 1). The analysis was carried out following an established protocol,^22^ and protein abundance across the samples was evaluated by comparing label-free quantification (LFQ) values obtained by MaxQuant.

**Table 1:** Relative Percentage of the First 20 Most Abundant Proteins (Calculated on the Average LFQ Values of the Triplicates)

Protein name	UniProt	MW (kDa)	STD (%)	S1 (%)	S2 (%)	U/T (%)
Alpha-2-HS-glycoprotein (Fetuin-A)	A0AAF6YGQ3	38	50.8	57.8	70.5	69.4
Albumin	P02769	69	12.1	10.4	1.3	1.9
Alpha-1-antiproteinase	P34955	46	10.6	9.0	15.2	8.3
Alpha-fetoprotein	Q3SZ57	69	3.1	3.0	0.2	x
Hemoglobin fetal subunit beta	P02081	16	2.6	3.0	1.9	3.1
Vitamin D-binding protein	F1N5M2	53	2.3	2.0	0.2	x
Interalpha-trypsin inhibitor heavy chain 4	F1MMD7	102	1.9	1.4	0.3	1.2
Complement C3	Q2UVX4	187	1.6	0.9	x	x
Angiotensinogen	P01017	51	1.6	1.4	x	0.5
Antithrombin-III	F1MSZ6	52	1.5	1.0	0.3	1.2
Serpin family G member 1	A0AAA9TZ37	53	1.5	1.2	0.2	x
Histone H2A	F2Z4F9	14	1.4	1.5	2.9	3.4
Alpha-2-macroglobulin	Q7SIH1	168	1.4	1.3	x	x
Interalpha-trypsin inhibitor HC2 component homologue	Q9TRI1	106	1.1	1.1	x	x
Complement C5	F1MY85	189	1.1	x	x	1.4
C4a anaphylatoxin	A0AAA9S3H4	193	1.1	0.7	x	x
Protein AMBP	F1MMK9	43	1.0	0.8	x	x
Serpin family D member 1	ENSBTAP00000018574	55	0.9	0.7	x	x
Apolipoprotein A-II	P81644	11	0.9	1.3	2.0	x
Fetuin-B	Q58D62	43	0.8	x	0.3	1.0
Pigment epithelium-derived factor	Q95121	46	0.7	0.8	x	0.4
Alpha-1B-glycoprotein	Q2KJF1	54	x	x	x	0.7
MEFV innate immunity regulator, pyrin	E1B7P5	64	x	x	0.4	x
Histone H2A type 2-C	A1A4R1	14	x	x	0.5	1.0
Interalpha-trypsin inhibitor heavy chain	P56652	100	x	x	0.4	0.4
Heat shock cognate 71 kDa protein	P19120	71	x	0.9	1.4	2.8
Actin, cytoplasmic 1	P60712	42	x	x	0.4	1.3
Heat shock protein HSP 90-beta	Q76LV1	83	x	x	x	0.5
Transthyretin	O46375	16	x	x	x	0.5
Thyroxine-binding globulin	G3MXX2	49	x	x	x	0.5
14-3-3 protein epsilon	P62261	29	x	x	x	0.4
Glyceraldehyde-3-phosphate dehydrogenase	P10096	36	x	x	x	x
DENN domain containing 4C	F1N032	216	x	x	0.4	x
Clusterin	P17697	51	x	x	0.8	x
Lumican	Q05443	39	x	x	0.4	x

**Figure 4:**
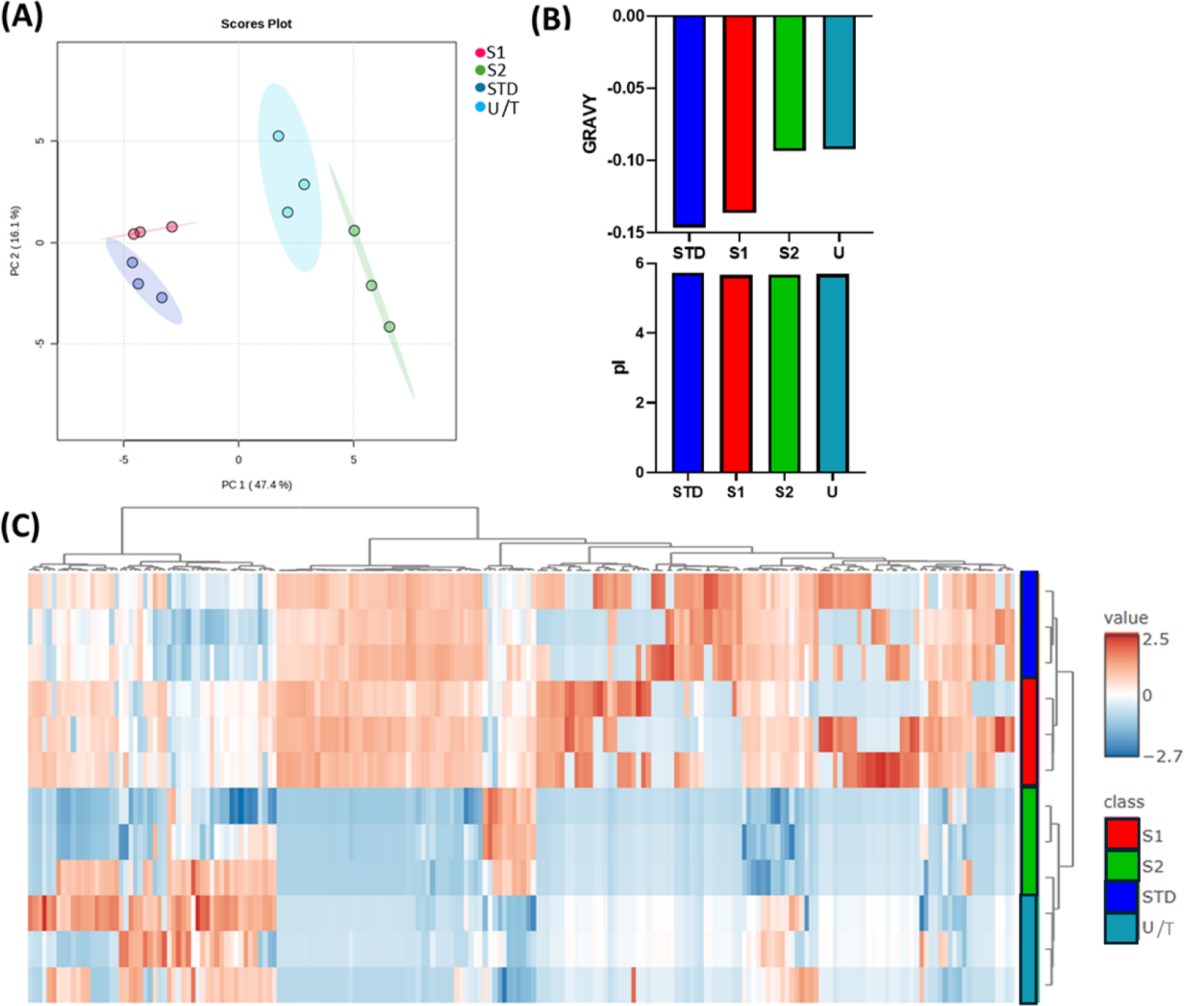
Statistical analysis of proteomics data from S1, S2, U/T fractions, and STD one of HC-GO1. (A) Principal Component Analysis (PCA) scores plot comparing the first and second principal components. The percentage of explained variance for each component is indicated in brackets. Each fraction was analyzed in triplicate, with each replicate represented as an individual dot. The 95% confidence ellipse for each group is depicted as a colored area. (B) average grand average of hydropathy (GRAVY) and isoelectric point (pI) values calculated for each sample group. (C) Hierarchical clustering visualized as a heatmap. The clustering was performed using Euclidean distance as the distance metric and Ward’s method (ward.D) as the clustering algorithm.

Table 1 shows the top 20 proteins in each fraction identified by mass spectrometry, which shows great similarities between STD and S1 and also between S2 and U/T. Indeed, both groups (STD–S1 and S2–U/T) share the same top three most abundant proteins, thus giving even more evidence of the correlation within each group.

Several proteins are consistently observed across all fractions, with Alpha-2-HS-glycoprotein (Fetuin-A) being the most abundant overall, while Albumin is instead the most abundant protein in FBS.^23^ Indeed, a lower bonding of Albumin to GO compared to other carbon-based materials was observed and attributed to the oxidation of the surface.^24^ Proteins like Albumin and Alpha-fetoprotein appear prominent in STD and S1, but they are less abundant in later elution steps (S2 or U), suggesting that they interact weakly with the surface or are more easily eluted. Conversely, proteins such as Histone H2A and Heat shock cognate 71 kDa protein emerge more predominantly in the S2 and U extractions.

The two abundant proteins, Albumin and Fetuin-A, contribute to the wide band that can be seen in the gel between 60 and 50 kDa. Indeed, even if the Alpha-2-HS-glycoprotein has a lower molecular weight based on the amino acid sequence, due to glycosylation, it migrates at a higher molecular weight.^25^ Moreover, the decrease of Albumin with subsequent elutions is consistent with densitometry analysis (Figure 3B) showing a decrease inside the broad band between 95 and 52 kDa of the band around 66 kDa in S2 and U/T, alongside an increase in the intensity of a band at a slightly higher molecular weight.

We further evaluated whether a correlation between the peptide hydrophobicity and the elution buffer of the biomolecular corona occurred by measuring the grand average of hydropathicity (GRAVY) values of the corona proteins. The GRAVY index establishes the hydrophobicity of a peptide by calculating the average hydropathy value, which is determined as the sum of the hydropathy values of all amino acids in the sequence divided by their length, where positive GRAVY values indicate hydrophobic peptides, while negative values infer hydrophilic ones.^26,27^ We considered the 20 most abundant proteins for each sample to calculate the average GRAVY parameter and the isoelectric point (pI) of the proteins in the corona (Figure 4B). Samples S1, S2, STD, and U show notable similarities in their pI values, which fall within a very narrow range of 5.66–5.72. However, there are discernible differences in the GRAVY values across the samples. The STD and S1 samples exhibit up to 33% more negative GRAVY values than S2 and U. This confirms the higher similarity between STD and S1 but also highlights that, during the elution, more resistant proteins are the most hydrophobic, while their charge seems to be irrelevant in determining the relative affinity of the protein for the GO surface.

PCA analysis was performed to assess and visualize differences in protein composition among the fractions (S1, S2, U/T) and the standard protocol (STD). The resulting score plot (Figure 4A) indicates a degree of similarity between STD and S1, as well as between S2 and U/T, based on their relative proximity. However, none of the fractions obtained using the modified protocol (S1, S2, U/T) fully overlap with the STD group, suggesting that they are not exactly comparable. This observation is further supported by the hierarchical clustering analysis shown in the heatmap (Figure 4C), where S1 clusters more closely with STD, and S2 groups with U/T, highlighting these intragroup similarities. This similarity between STD–S1 and S2–U/T is also supported by pairwise comparisons (Figure S4), where these are the only sample pairs that did not show any significant fold changes in protein intensities.

### X-ray Photoelectron Spectroscopy (XPS) Analysis

XP spectra were acquired for the pellets remaining after applying both the STD and modified elution protocols to HC-GO1, as well as for GO1 untreated after applying the modified protocol (BK) (Figures S5 and S6). Table S2 presents the atomic compositions of all materials involved in the protocol. The presence of Si in STD is a clear indication of the presence of urea, as it is the only component that displays trace amounts of Si. The minuscule presence of Cu in the urea sample can be attributed to the catalytic process that produces urea (copper dust/mist/fume), which is highly dependent on the catalyst type: Monel catalyst (31.5% Cu, 66.5% Ni).^28^ While full thorough XP spectra peak deconvolution is lacking, it is possible to attribute the set of peaks around 163 eV to carbon–sulfur bonding environments and the other set of peaks around 169 eV to sulfur oxides in the S 2p high-resolution XP spectra.^29^ The latter species exist in GO1, due to the chemical oxidation process, wherein covalent sulfates emerge on GO1 basal planes. The comparison of the shapes of S 2p spectra reveals that thiourea characteristic bonding is dominant in BK while STD retains a sizable amount of SDS, but most importantly, BK preferentially uptakes thiourea more than SDS.

## Discussion

The results reported in this study point out that the physicochemical properties of Ad-NMs, such as GO, pose special challenges to the conventional protocols of protein elution and corona characterization given its strong interaction with proteins.^30^ Indeed, our findings show that the standard elution protocol is unable to completely desorb proteins from GO. The STD protocol (SDS-based) has been extensively used for the characterization of GO protein corona.^31–36^ Elution from the GO pellet is crucial because, depending on the approach, it is followed either by the load on the gel of both the pellet and buffer, which is variable, or by centrifuging the sample and loading only the eluate. Moreover, the need for the complete removal of proteins from the pellet is even more evident when considering that extractions performed at different SDS concentrations on HC-GO1 result in varying protein profiles. SDS binds to amino acids, allowing for protein unfolding and the diffusion of a uniform negative charge. This negative charge acquired by proteins allows the detachment from the NMs.^37^ However, in the case of stronger interactions, chaotropic compounds can help the extraction of proteins by disrupting both hydrogen bonds and hydrophilic interactions. Moreover, when used at high concentrations, they are capable of destroying secondary protein structures, helping solubilization.^38^ Therefore, we developed an improved elution protocol, which combined the application of chaotropic agents, urea and thiourea^21^ along with STD buffer-based extraction. The application of this stepwise extraction procedure indeed increased the recoveries substantially, resulting in only a minor amount of proteins remaining on GO surfaces. The presence of residual proteins in the pellet was evaluated by SDS-PAGE using Coomassie Brilliant Blue staining to visualize the protein band intensity, allowing relative quantification. Although more sensitive silver staining can reveal a lower protein content, the trace proteins detected by this method would be totally negligible compared to the prominent bands observed with Coomassie staining. Differences between proteins eluted according to STD and modified protocols were also revealed by protein analysis, in particular, by mass spectrometry. Indeed, although S1 shows a composition very similar to STD, subsequent fractions S2 and U extracted proteins with different features, including higher hydrophobicity, expressed by their GRAVY values. It would thus appear that the hydrophobic proteins strongly adsorbed onto the GO surface are unable to be removed by STD extraction protocols. This is also confirmed by XPS analysis, which reveals the stronger retention of thiourea on GO compared with urea, which can be attributed to differences in their molecular interactions with the GO surface. GO possesses hydrophilic functional groups (e.g., hydroxyl, epoxy, and carboxyl groups) and hydrophobic graphitic regions, enabling van der Waals forces and π–π stacking.^30,39^ Urea, being polar due to its C=O and NH_2_ groups, primarily forms hydrogen bonds with the hydrophilic regions of GO. In contrast, with its C=S group, thiourea is less polar and more hydrophobic, allowing it to interact more effectively with GO hydrophobic graphitic regions via van der Waals forces and π–π stacking. While urea relies predominantly on hydrogen bonding, which can be disrupted during aqueous elution, thiourea dual ability to form hydrophilic and hydrophobic interactions enables it to remain bonded to GO. As previously discussed, the protein composition of the S2 and U fractions corresponds to more hydrophobic proteins; therefore, it is possible that excess thiourea, added in the last buffer, breaks hydrophobic interactions and replaces these proteins on GO surfaces. These findings align with previous reports in the literature. Quagliarini et al. investigated the displacement of protein from GO by DNA, revealing that proteins are displaced with increasing DNA concentrations, but certain proteins have shown resistance, which could be attributed to their strong affinity for the GO surface.^40^ Moreover, Lu et al. studied how protein properties influence interaction with the GO surface, highlighting the role of hydrophobic interactions in the formation of the protein corona. Indeed, it has been demonstrated that simple electrostatic forces are not the primary drivers of protein interactions with the GO surface, as evidenced by the lack of variation in the pI across different extractions. Instead, hydrophobic residues appear to play a central role, with their adsorption occurring following initial hydrophilic interactions.^41^ This sequence suggests that hydrophilic adsorption triggers structural modifications in proteins, leading to the exposure of hydrophobic regions, which subsequently enhances their interaction with the surface. The degree of oxidation on GO surfaces influences protein adsorption behavior by increasing the number of hydrophilic groups available for interaction; however, this effect is not uniform across all proteins. For instance, the contact surface area of human serum albumin and IgE is primarily influenced by hydrophobic residue adsorption, decreasing as surface oxidation increases. In contrast, the contact surface area of ApoE remained unaffected by surface hydrophilicity. Protein flexibility also plays a significant role, as more flexible proteins are more prone to structural modifications that expose hydrophobic regions. Additionally, the spatial distribution of hydrophobic and hydrophilic domains within a protein further affects the interaction strength.^41^ Therefore, the strength and nature of interactions of proteins with GO are highly protein-specific. This underscores the importance of implementing protocols capable of characterizing the complete set of interactions to gain a comprehensive understanding of the protein corona. Indeed, the SDS-PAGE profiles of the pooled S1, S2, and U/T aliquots suggest the presence of differences among the three different GOs. Although studying the influence of physicochemical properties on the corona was not the primary aim of this study, these findings support the potential impact of such characteristics.

## Conclusion

The present results have an impact that extends beyond the methodological improvements. The modified protocol recovers a wider range of proteins, including those bound with stronger affinity, thus being more representative of the biomolecular corona. This is important for the critical assessment of biological interactions and eventual hazards related to GO and other Ad-NMs. Incompletely desorbed proteins upon STD protocols that are still used today may yield a very underestimated view of protein corona complexity, leading to a biased interpretation of the nanomaterial behavior in a biological environment. In addition, our study underlines the importance of protocol standardization and optimization with regard to the specific properties of different Ad-NMs. The research area of nanosafety heavily relies on robust, reproducible methods for describing nano–bio interactions. Ad-NMs are increasingly transferred from laboratory research into industrial applications; thus, the establishment of reliable protocols is required for their safe and sustainable incorporation into consumer products.

In summary, this work outlines the limitations of the existing protein elution methods for GO and presents an effective alternative protocol that allows for complete protein recovery. Such tailored approaches are essential to further our understanding of biomolecular coronas and guarantee the proper assessment of the nanomaterial safety and functionality. Future studies with GO should aim to further use and refine the protocol presented here, enabling a comprehensive understanding of how different characteristics of GO (e.g., oxidation) influence the resulting corona. Moreover, it is likely that other carbon materials, such as carbon nanotubes, exhibit similarly high surface reactivity and strong interactions with proteins. This necessitates the development of tailored protocols for which the presented method can serve as a valuable starting point, enabling a more thorough investigation of nano–bio interactions.

## Materials and Methods

### Materials

GO samples were obtained from Graphenea. Thiourea was purchased from BDH Chemicals (30423). The following reagents were purchased from Sigma-Aldrich: urea (U5128), Tris base (99%), acrylamide/bis-acrylamide 40% solution, sodium dodecyl sulfate (SDS, 99%), ammonium persulfate, *N*,*N*,*N*,*N*-tetramethylethylenediamine (TEMED, 99.5%), phosphate-buffered saline (PBS) tablets, d-(+)-sucrose (99.9%), and glycine (Sigma, G8898). One PBS tablet was dissolved in 200 mL of ultrapure water to obtain a 0.01 M phosphate buffer, 0.0027 M potassium chloride, and 0.137 M sodium chloride solution (pH 7.4 at 25 °C). 3X Blue Loading Buffer and 30X reducing agent (1.25 M DTT) were purchased from Cell Signaling Technology (USA). The Prime-Step Prestained Protein Ladder was purchased from BioLegend (Ireland). Imperial Protein Stain and Pierce C18 Tips were purchased from Thermo Scientific Ireland. Lacey carbon Cu grids were purchased from Ted Pella Inc., USA.

### Characterization of GO Samples

For the transmission electron microscopy (TEM) studies, all GO materials were dispersed in ethanol. Before drop-casting on lacey carbon Cu grids, the dispersions were subjected to soft sonication until they became homogeneous. The grids were left to dry under ambient conditions prior to investigation. TEM experiments were conducted on a JEOL JEM-2100F UHR 200 kV electron microscope (Tokyo, Japan). Scanning electron microscopy (SEM) was conducted on a JEOL SEM JSM-IT800HL (Tokyo, Japan) equipped with an energy-dispersive X-ray spectroscopy (EDS) system, ULTIM EXTREME SEM, with AZTEC software from OXFORD INSTRUMENTS PLC & SUBSIDIARIES (Abingdon, UK). Samples were prepared in Milli-Q water (0.05 g/L) and drop-casted (10 μL) on a Si wafer chip. Atomic force microscopy (AFM) measurements were conducted on a Bruker MultiMode 8 atomic force microscope. AFM sample preparation was identical to SEM sample preparation.

### GO-Hard Corona Isolation Protocol

Protein corona complexes of GO were obtained by incubating 0.5 mg/mL of GO in 0.5 mL of 10% FBS in PBS. After incubation (37 °C, 1 h, 300 rpm), the samples were centrifuged at 10 000 g for 5 min. Then, the supernatant was carefully removed without disturbing the pellet, 0.5 mL of PBS was added, and finally, the pellet was resuspended by 30 s of vortex. Indeed, the pellet is generally redispersed by pipetting,^22^ but in the case of GO, this results in the adsorption of particles on the tip, resulting in sample loss. The samples were then subjected to centrifuge-redispersion cycles another three times, obtaining the HC-GO.

### Protocol for HC Elution from GO and Analysis

#### Standard Protein Corona Elution Protocol (STD Protocol)

The standard protocol for protein elution was carried out following an established protocol developed in the group^22^ (Figure 2A). Briefly, after the HC-GO samples were prepared , theywere resuspended with the STD elution buffer by adding 12 μL of water with 6 μL of 3X blue loading buffer to reach a final composition of the standard buffer of 62.5 mM Tris (6.8 pH), 2% w/v sodium dodecyl sulfate (SDS), 10% w/v glycerol, 0.01% bromophenol blue (BPB), and 41.7 mM DTT (Table S3). The samples were then incubated for 5 min at 95 °C and centrifuged for 3 min at 18 000 rcf in order to pellet the GO while the eluted corona proteins remained in the supernatant and were processed for additional analysis.

#### Modified Protein Corona Elution Protocol (Modified Protocol)

In the modified extraction protocol, 50 μL of the STD buffer is added to the HC-GO pellet, and samples are shaken (1500 rpm) at 95 °C for 5 min. Then, samples are centrifuged (18 000 g, 3 min), the supernatant is collected (S1), 50 μL of fresh STD buffer is added to the pellet, and then, the incubation and centrifugation steps are repeated. After centrifugation, the second aliquot of STD buffer (S2) is collected, and 50 μL of urea/thiourea buffer (U/T) is added to the pellet and maintained for 2 h under continuous shaking (1500 rpm) at room temperature. Indeed, heating is avoided to minimize the risk of carbamylation, which can occur when proteins are in aqueous urea solutions, particularly at elevated temperatures. The U/T buffer composition is equivalent to that of the STD buffer but with the addition of 6 M urea and 2 M thiourea (Table S3). After 2 h of incubation, samples are centrifuged, and the last extraction aliquot is collected (U/T). The workflow of the procedure is reported in Figure 2B.

#### Protocol for SDS-PAGE Analysis of Eluted Protein Corona

SDS-PAGE was performed with a 10% acrylamide gel prepared as previously reported.^22^ Different sample volumes were loaded into the gel wells to adjust the protein amount, allowing the band visualization, and run at 120 V in the presence of running buffer (glycine 1.44%, SDS 0.10%, Tris 0.303%). After the gel run, protein bands were visualized by Coomassie Brilliant Blue staining following the manufacturer’s instructions. Densitometry was obtained by GelAnalyzer 19.1 (www.gelanalyzer.com, created by Istvan Lazar Jr., PhD and Istvan Lazar Sr., PhD, CSc).

#### Protocol for Mass Spectrometry Analysis

Mass spectrometry samples were prepared in biological triplicates following previously established protocols.^22^ The samples of the isolated HC-GO were run on the SDS-PAGE. Loaded volumes corresponded to 3 μL for STD and S1 and 20 μL for S2 and U/T. Samples were run until the blue front reached the mark at 0.5 cm below the line between the separating and stacking gels. All of the area in the gels between that line and the blue front, which contained the protein, was cut and placed in a centrifuge tube and processed for further analysis. The proteins were then fixed, in-gel reduced, and alkylated before digestion with trypsin overnight at 37 °C (16 h). Subsequently, the gel pieces were subjected to peptide digestion, and the peptide digestion products were recovered and washed with C18 tips according to the manufacturer’s protocol. The amount of digested peptides from each sample was quantified by a NanoDrop ND-2000 before the mass spectrometry analysis. Analysis was performed using liquid chromatography-tandem mass spectrometry (LC-MS/MS)­on a Dionex UltiMate 3000 nanoRSLC coupled in-line with an Orbitrap Fusion Tribrid mass spectrometer (Thermo Fisher, Ireland). In particular, LC-MS run time was 60 min, using data-dependent acquisition, full MS scan in the Orbitrap at 120 K resolution, and MS/MS with HCD in the Orbitrap at 15 K resolution. Data were then analyzed with MaxQuant (v. 2.6.7.0).^42^ The MS/MS spectra were searched using the Andromeda search engine against forward and reversed UniProt *Bos taurus* (Bovine) (proteome ID UP000009136). Cysteine carbamidomethylation was set as a fixed modification, while N-terminal acetylation and methionine oxidation were variable modifications. Protein and peptide identifications in the present study used a 0.01 FDR threshold at both the protein and peptide levels, considering only peptides of amino-acid length seven or more. A standard target-decoy database approach was applied for other major search filtrations. Other major search parameters included an MS/MS mass tolerance of 0.02 Da, a peptide mass tolerance of 10 ppm, and tolerance for the occurrence of up to two missed cleavages. The LFQ was restricted to proteins identified with at least two unique peptides. Statistical analysis was performed on the online platform MetaboAnalyst (a web-based comprehensive platform for metabolomics data analysis, developed by Xia Lab, Montreal, Canada) and Python (version 3.12.2) and the Pandas library (version 2.2.2), implemented via the Anaconda distribution (Anaconda Inc., Austin, TX, USA). Prior to statistical analysis, the data were normalized by sum, log10-transformed, and mean-centered. Both hierarchical clustering dendrogram and heatmap used the Euclidean distance measure and Ward clustering algorithm. The GRAVY and pI were calculated for the 20 most abundant proteins in each sample. GRAVY was measured by Kyte-Doolittle and Hopp-Woods formulas.^27^ These values represent a weighted average based on the LFQ values, providing insights into the overall hydrophilicity and charge characteristics of the proteins.

### XPS Analysis

HC-GO subjected to STD and modified extraction protocols were analyzed by XPS in order to estimate the amount of proteins remaining in the pellet. The modified protocol was also applied to the GO1 pellets, derived from the same incubation step of the HC-GO but obtained in the absence of proteins in order to get the blank signal (BK). Six different pellets for each condition were pooled together. XP spectra were recorded using a PHI XPS VersaProbe III energy spectrometer equipped with a monochromatic 1486.6 eV Al–Kα radiation source. A focused X-ray source with an X-ray beam size of 100 μm, a power of 25 W, and an e-beam energy of 15 kV was used. Charge neutralization was possible using a complementary dual-beam charge neutralization method. Three remote spots on each sample were used to acquire survey spectra. The C 1s peak at 284.8 eV was used as a reference to calibrate all acquired spectra.

## Supplementary Material



## Data Availability

The mass spectrometry data underlying this study are openly available in Zenodo (https://doi.org/10.5281/zenodo.15807118).

## Notes

Additional SDS-PAGE analyses and related protocol development details, pairwise analysis of proteomics data, extended XPS data, and buffer compositions (PDF)
