# Supplementary material for: A Redefined Protocol for Protein Corona Analysis on Graphene Oxide
Source: ACS Nanosci Au. 2025 Jul 24;5(5):388–97. doi: 10.1021/acsnanoscienceau.5c00052 (PMC12532052; doi:10.1021/acsnanoscienceau.5c00052)
Supplement: Supplementary file 1 [file ng5c00052_si_001.pdf]

Supporting Information

A Redefined Protocol for Protein Corona Analysis on Graphene Oxide

Asia Saorin[1], Ahmed Subrati[2], Alberto Martinez-Serra[1], Beatriz Alonso[3], Michael Henry[4], Paula Meleady[4], Sergio E. Moya[2], Marco P. Monopoli[1*]

[1] Department of Chemistry, Royal College of Surgeons in Ireland (RCSI), Dublin, D02 YN77, Ireland
[2] Center for Cooperative Research in Biomaterials (CIC biomaGUNE), Basque Research and Technology Alliance (BRTA), San Sebastian, 20014 Spain
[3] GRAPHENEA SA, San Sebastian, 20009, Spain
[4] School of biotechnology, Dublin City University (DCU), Dublin, D09 W6Y4, Ireland

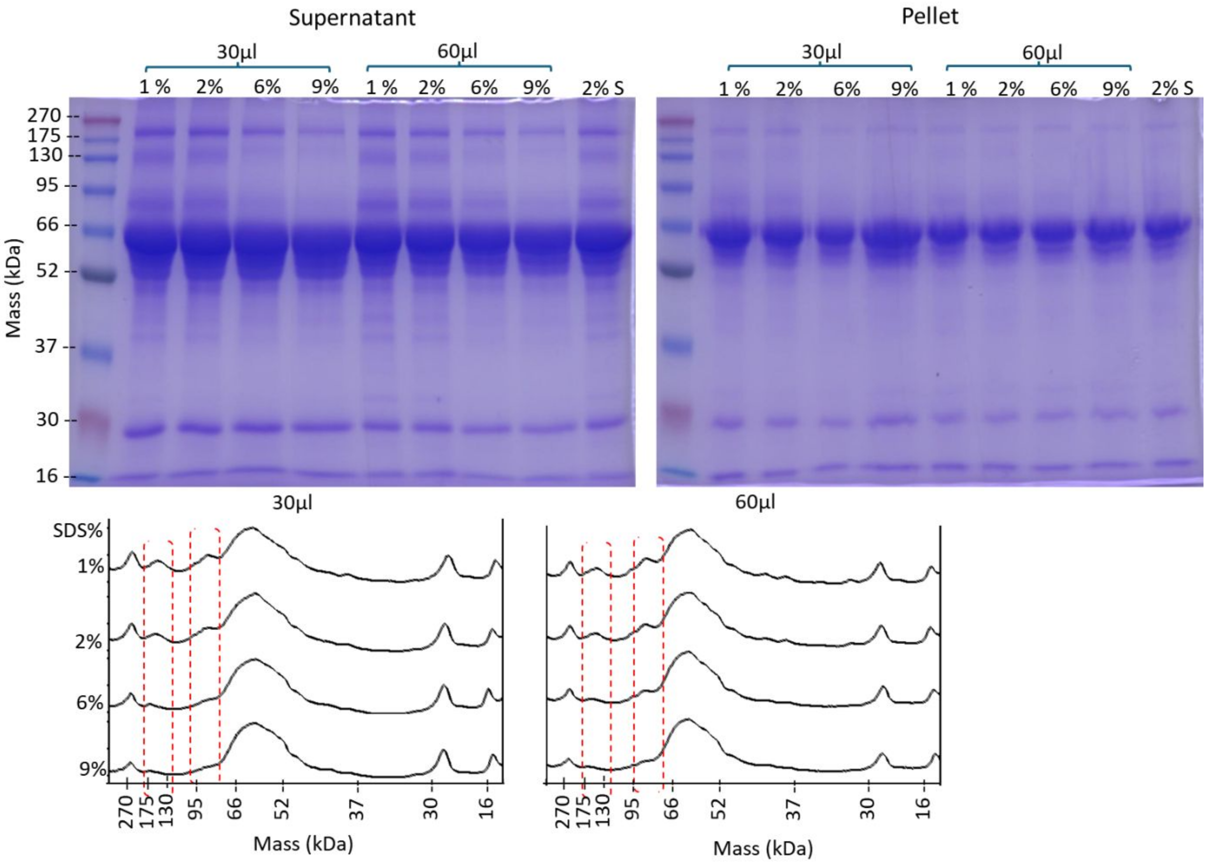

Figure S1. HC-GO1s were subjected to standard elution protocol but with different amounts of SDS in the loading buffer (1-9%), different buffer volumes added for the elution (STD-B 30 and 60 µL) and after the addition of 5 minutes of bath sonication in the presence of 2% SDS (2%S). On the top gel images, on the bottom gel densitometry of supernatant profiles where the bands that are affected by SDS concentration are highlighted.

Table S1. Description of the different tested samples for extraction protocol optimization. HC pellets of GO1 were subjected to extraction with different volumes of standard buffer (STD-B) and urea buffer (U-B). For comparison, the standard protocol (STD) was applied. After the addition of STD-B, samples were incubated for 5 minutes at 95 °C, as reported for the standard protocol. After the addition of U-B, samples were shaken (1500 rpm) at room temperature for 2 hours. After each extraction, samples were centrifuged (18,000g, 3 minutes), and the supernatant was collected while the pellet was subjected to the following extraction step.

	**STD buffer µl (aliquot)**		**U/T buffer µl (aliquot)**
**sample name**	**elution1**	**elution2**	
**T1**	100 (S1)	x	50 (U/T)
**T2**	50 (S1)	50 (S2)	50 (U/T)
**T3**	50 (S1)	50 (S2)	x
**T4**	150 (S1)	x	50 (U/T)
**STD**	18 (STD)	x	x

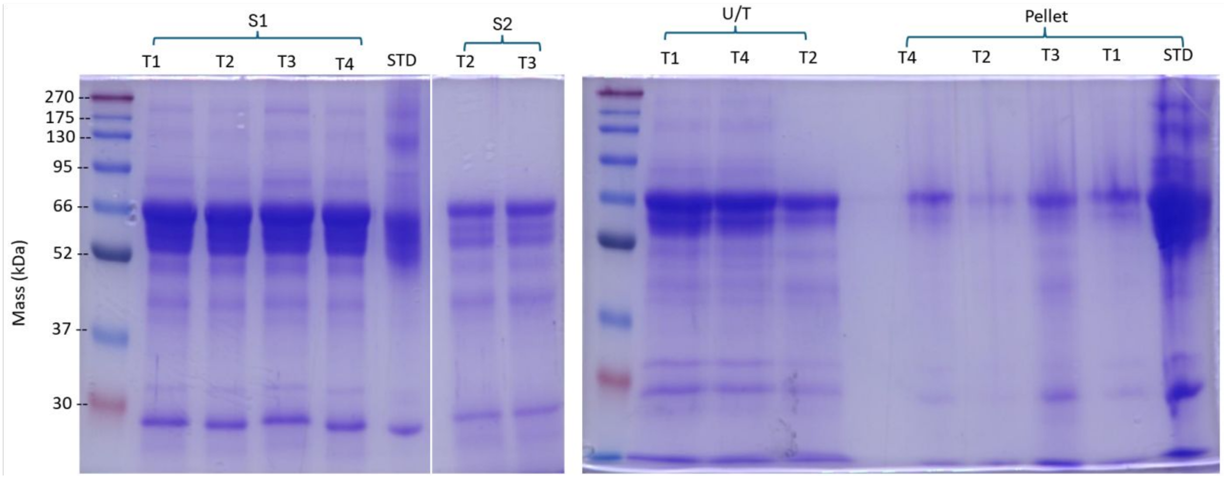

Figure S2. SDS-PAGE analysis of HC-GO1 subjected to different extraction procedures reported in Table S2. Loaded volumes were adjusted based on the added buffer volumes and protein content. This corresponded to 0.72 µL for STD; for S1, loading volumes were 3 µL for T2 and T3, 6 µL for T1, and 9 µL for T4. For S2 and U/T, loading volumes were 10 µL and 20 µL, respectively.

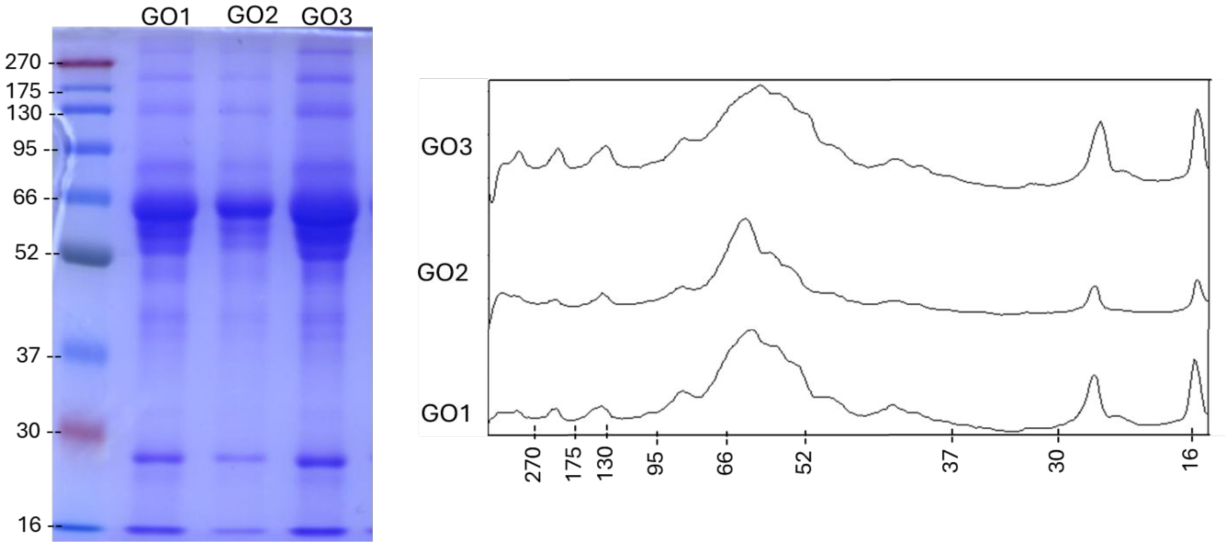

Figure S3. SDS-PAGE analysis of HC-GOs following the modified elution protocol. Collected fractions (S1, S2, and U/T) were pooled and then 9 μL were loaded.

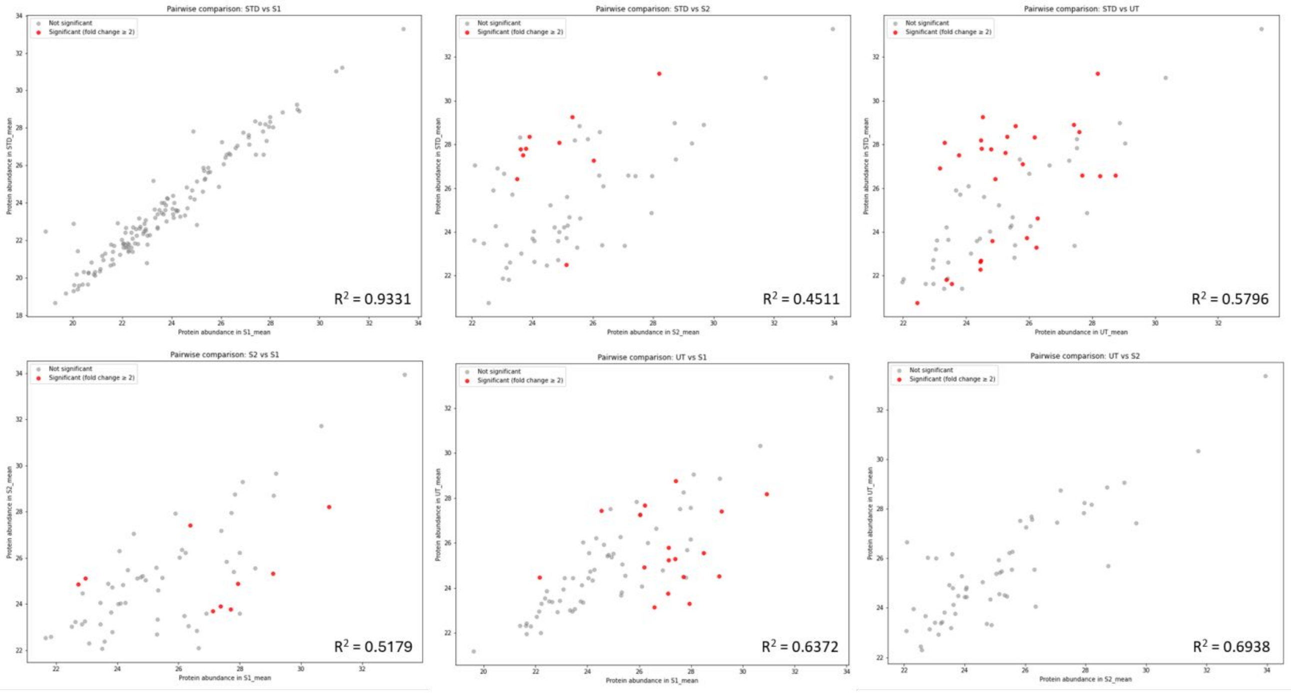

Figure S4. Pairwise comparison of LFQ values between fractions obtained using the modified protocol (S1, S2, U/T) and the standard (STD) protocol. Proteins showing at least a twofold change in abundance are highlighted.

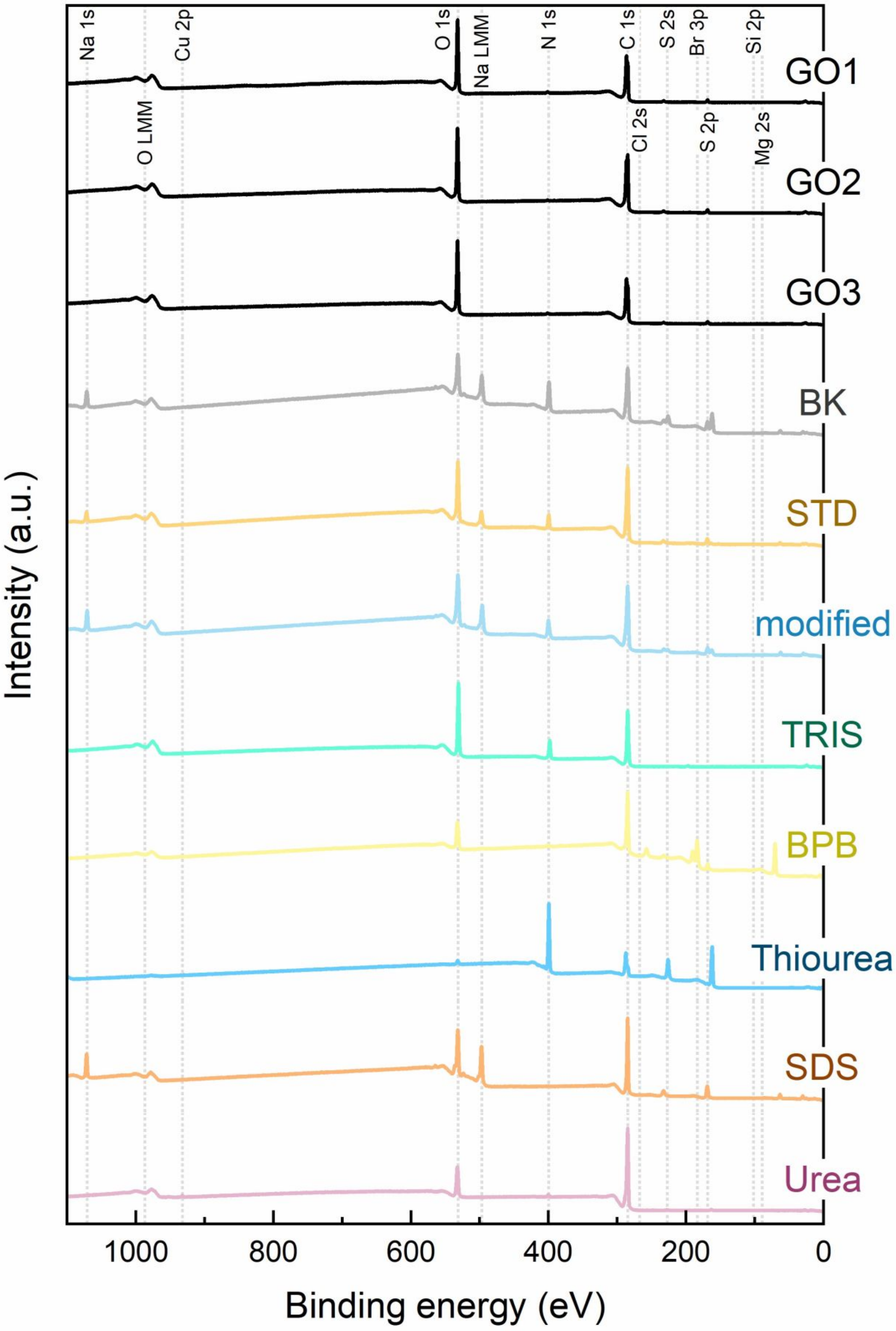

Figure S5. X-ray photoelectron survey spectra of all materials involved in the protocol. XPS analysis was conducted on the residual pellets obtained following the application of both the standard (STD) and modified elution protocols to HC-GO1, as well as on untreated GO1 subjected to the modified protocol (BK). For comparison the analysis is reported also for all pristine GOs.

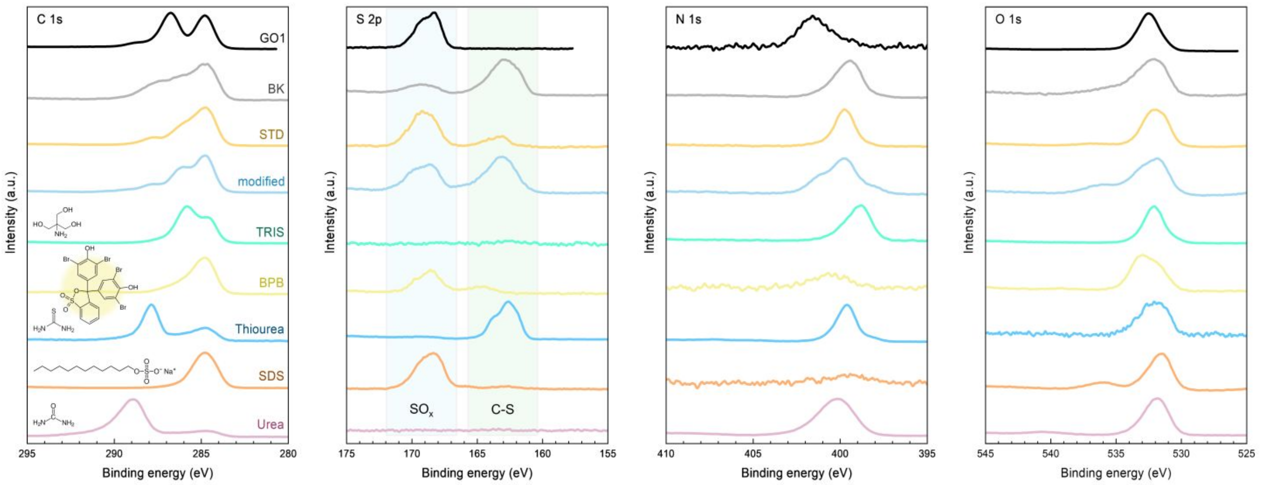

Figure S6. X-ray photoelectron high-resolution spectra of all materials involved in the protocol. XPS analysis was conducted on the residual pellets obtained following the application of both the standard (STD) and modified elution protocols to HC-GO1, as well as on untreated GO1 subjected to the modified protocol (BK). Analysis was also performed on pristine GO1.

Table S2. Elemental quantification by wide scan survey XPS of all materials involved in the protocol.

Sample	Composition (at.%)										Total
	C	O	N	Na	S	Si	Mg	Cl	Br	Cu	
GO1	70.20	27.70	0.70	-	1.40	-	-	-	-	-	100.00
GO2	75.08	23.02	0.39	-	1.51	-	-	-	-	-	100.00
GO3	70.62	27.50	0.53	-	1.35	-	-	-	-	-	100.00
modified	65.72	18.73	8.15	1.50	5.90	-	-	-	-	-	100.00
BK	55.93	16.51	13.09	1.74	12.73	-	-	-	-	-	100.00
STD	70.81	19.09	5.32	0.94	3.23	0.61	-	-	-	-	100.00
TRIS	63.66	25.45	10.52	-	-	-	0.14	0.24	-	-	100.00
SDS	69.39	20.23	-	2.70	7.68	-	-	-	-	-	100.00
BPB	71.29	12.51	1.49	-	4.20	-	-	-	10.51	-	100.00
Thiourea	25.49	1.38	47.77	-	25.37	-	-	-	-	-	100.00
Urea	85.78	11.80	1.36	-	0.58	0.34	0.05	-	-	0.09	100.00

Table S3. Composition of buffers. Blue loading buffer is used for the preparation of standard buffer by dilution 1:3 with water and for the preparation of urea/thiourea buffer by dilution 1:3 with urea/thiourea (9M/3M) water solution.

	3x Blue loading buffer	STD buffer	U/T buffer
Tris pH6.8 (mM)	187.5	62.5	62.5
SDS (%wt/vol)	6	2	2
glycerol (%wt/vol)	30	10	10
BBP (%wt/vol)	0.03	0.01	0.01
DTT (mM)	1.25M	41.7	41.7
Urea (M)	x	x	6
Thiourea (M)	x	x	2
